# Get Phases from Arsenic Anomalous Scattering: *de novo* SAD Phasing of Two Protein Structures Crystallized in Cacodylate Buffer

**DOI:** 10.1371/journal.pone.0024227

**Published:** 2011-09-02

**Authors:** Xiang Liu, Heng Zhang, Xiao-Jun Wang, Lan-Fen Li, Xiao-Dong Su

**Affiliations:** State Key Laboratory of Protein and Plant Gene Research and Biodynamic Optical Imaging Center (BIOPIC), School of Life Sciences, Peking University, Beijing, China; Institut Pasteur, France

## Abstract

The crystal structures of two proteins, a putative pyrazinamidase/nicotinamidase from the dental pathogen *Streptococcus mutans* (*Sm*PncA) and the human caspase-6 (Casp6), were solved by *de novo* arsenic single-wavelength anomalous diffraction (As-SAD) phasing method. Arsenic (As), an uncommonly used element in SAD phasing, was covalently introduced into proteins by cacodylic acid, the buffering agent in the crystallization reservoirs. In *Sm*PncA, the only cysteine was bound to dimethylarsinoyl, which is a pentavalent arsenic group (As (V)). This arsenic atom and a protein-bound zinc atom both generated anomalous signals. The predominant contribution, however, was from the As anomalous signals, which were sufficient to phase the *Sm*PncA structure alone. In Casp6, four cysteines were found to bind cacodyl, a trivalent arsenic group (As (III)), in the presence of the reducing agent, dithiothreitol (DTT), and arsenic atoms were the only anomalous scatterers for SAD phasing. Analyses and discussion of these two As-SAD phasing examples and comparison of As with other traditional heavy atoms that generate anomalous signals, together with a few arsenic-based *de novo* phasing cases reported previously strongly suggest that As is an ideal anomalous scatterer for SAD phasing in protein crystallography.

## Introduction

SAD (single-wavelength anomalous diffraction) is a widely used phasing method in protein crystallography due to its advantages such as single dataset collection, minor radiation damage, and no need for maximum anomalous effects etc. [1]. In the recent years, synchrotron radiation (SR) X-ray sources have been established all over the world [2]. Given SR's advantageous features such as extremely high intensity and stability and tunable wavelength selection, SR facilities have become the most favorable choices for diffraction data collection for protein crystallography. The statistics from PDB (protein data bank) database shows that nearly 80% of protein crystallographic diffraction data were collected on SR facilities so far (http://biosync.sbkb.org/index.jsp, until 2010). On the other hand, new software packages for data processing, phasing, density modification and model auto-building increase the efficiency and throughput in SAD phasing [3]. These technical progresses have made the SAD method the most convenient and powerful phasing method in protein crystallography.

The anomalous scatterers in SAD phasing method can be atoms inherited in native proteins (e.g. Zn, Fe, Cu in metalloproteins [1] or S from cysteines and methionines [4], [5]); or artificially introduced into proteins by soaking (i.e. heavy atoms such as Hg, Au, Pt, halide salts [6], [7] and lanthanide [8], [9]); or genetically incorporated (Selenomethionine (Se-Met) substitution [10] and lanthanide binding tags (LTB-tags) fusion for proteins [11]); or chemically synthesized (bromouracyl/bromocytosin substitution for nucleic acids [12], [13]). Heavy-atom or halide soaking method requires extra efforts for the screenings of soaked compounds, and the experimental conditions can only be obtained by trial and error. Moreover, soaking procedures may bring in considerable disturbances to the protein structure and the crystal lattice, resulting in the decrease of the diffraction resolution. By contrast, the Se-Met substitution method has been more extensively used due to its definite incorporation protocol and high success rate [14]. The first Se-Met labeled protein structure was reported in 1990 [10]. Till August 2010, more than 5000 Se-Met labeled crystal structures have been deposited in the PDB database (http://www.pdb.org/pdb/home/home.do, August 2010, PDB statistics,). Increasing number of newly-built synchrotron beamlines set their optimal wavelengths around the selenium *K* absorption edge (∼0.98 Å) [15] and nearly half of the structures solved by SAD phasing method took selenium as the anomalous scatterer [14].

**Figure 1:**
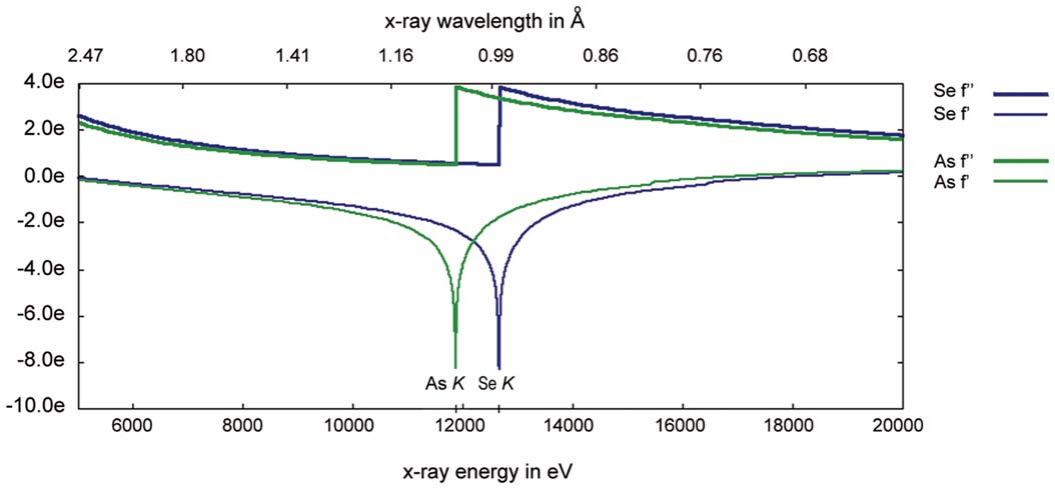
The plot of theoretical f prime and f double prime for arsenic and selenium. In this plot, the lines of arsenic are in green while the lines of selenium are in blue. The plot is generated from the following website: http://skuld.bmsc.washington.edu/scatter/.

Arsenic (As) is located beside selenium in the periodic table with a *K* absorption edge (∼1.04 Å) close to that of Se (∼0.98 Å). This feature suggests that users can collect As anomalous signals on an Se-dedicated SR beamline (Fig. 1). Till now, more than 200 structures with arsenic atoms presented in their coordinates in the PDB database can be found, but only three protein structures have been reported to use As as the anomalous scatterers for phasing [15], [16], [17]. The reasons that As have been neglected for long as a general scatterer in SAD phasing method are presumably due to its toxicity [19], [20] and a lack of general protocols for As incorporation. In fact, arsenic compound is toxic because it can interact with thiol-groups in biological macromolecules. Making use of this reaction [21], however, we can covalently introduce As into protein samples.

In this paper, we have made in depth analyses to evaluate the incorporation of arsenic atom into proteins and the general application of As as a common anomalous scatterer. We present two cases of protein structures determined by As-SAD phasing in our laboratory during the last two years, a putative PncA from *Streptococcus mutans* and caspase-6 from *Homo sapiens*. The crystallization conditions of both proteins contained cacodylate buffer, which specifically modified surface cysteines of the proteins. Both datasets were collected on SR facilities at the wavelength of 1.000 Å, the high-energy end from the *K* absorption edge of As (∼ 1.04 Å), and sufficient anomalous signals for SAD phasing were obtained. Our current studies demonstrated that cacodylate buffer is an effective reagent to covalently modify protein surface cysteines, and that As could serve as an ideal anomalous scatterer in SAD phasing method.

## Materials and Methods

### *Sm*PncA

#### Protein preparation

*Sm*PncA gene was cloned into the pET28a expression vector with an N-terminal (His)_6_-tag, and over-expressed in *Escherichia coli* BL21(DE3) strain at 30°C for 8 hours. The *Sm*PncA proteins were purified in two steps with a nickel chelating column first (5 ml HiTrap HP column, GE Healthcare/Amersham), followed by a gel filtration column (120 ml Superdex-75, GE Healthcare/Amersham). The supernatant of cell lysate was loaded on a nickel column equilibrated in buffer containing 20 mM Tris-HCl pH 7.5, 500 mM NaCl. The loaded column was then washed with equilibration buffer containing 100 mM imidazole, and the target proteins were eluted with a linear gradient with the equilibration buffer containing 500 mM imidazole. The proteins were further purified by gel filtration in buffer containing 20 mM Tris-HCl pH 7.5 and 200 mM NaCl. Purified proteins were concentrated for crystallization using the Amicon Ultra Centrifugal Filter Devices of 10,000 MWCO (Millipore Corporation). The concentration of the purified *Sm*PncA was about 20 mg/ml.

#### Crystallization and data collection

Crystals of *Sm*PncA were grown in 20% v/v 2-methyl-2,4-pentanediol (MPD), 0.1 M sodium cacodylate pH 4.5, and crystals suitable for data collection were obtained in half a month. Diffraction data were collected on the Beamline BL5A at the Photon Factory, Tsukuba, Japan. The crystal was flash frozen in liquid nitrogen and then maintained on the goniometer at 100 K in a stream of cold nitrogen. The diffraction data were collected from one single crystal at wavelength of 1.000 Å, and 360 frames were collected with 1° oscillation per image. The data were indexed, integrated and scaled by the program XDS [22], [23]. The statistics of the data are summarized in Table 1.

**Table 1:** Data collection and Statistics from crystallographic analyses.

	*Sm*PncA	Ac-VEID-CHO inhibited Casp6
Wavelength (Å)	1.000	1.000
Space group	*P* 2_1_2_1_2_1_	*P* 2_1_
Cell dimension		
a, b, c (Å)	76.5, 80.1, 130.9	55.4, 89.5, 61.1
		β = 111.70
Resolution (Å)	50.0–1.6 (1.7–1.6)	25.30–1.63 (1.71–1.63)
*R*_sym_ a (%)	5.6(68.2)	8.3(32.9)
Mean *I*/σ*I*	26.9(4.4)	7.0 (2.2)
Completeness (%)	99.5 (100.0)	99.1 (99.4)
Redundancy	13.6	2.2
Anomalous completeness (%)	99.5 (100.0)	72.4(68.1)
Anomalous redundancy	7.1	1.3
Refinement		
Resolution range (Å)	20.0–1.7	20.00–1.63
Number of reflections	88570	68845
*R*_work_ b/*R*_free_ b c (%)	16.03/ 19.12	16.30/ 19.37
Average *B*-factors	22.52	10.53
No of protein residues	727	480
No of waters	598	681
No of ligands	8	11
*rms*. *deviation* d		
Bond lengths (Å)	0.009	0.007
Bond angles (°)	1.164	1.098

Values in parentheses are for the highest resolution shell.

a *R*_sym_  = ∑|*I*_obs__*I*_avg_|/∑*I*_obs_.

b *R*_work, free_  = ∑||*F*_obs_|_|*F*_calc_|| / ∑|*F*_obs_|.

c *R*_free_ values are calculated for a randomly selected 5% of the data that was excluded from the refinement.

d Root mean square deviation from ideal/target geometries.

#### SAD phasing and refinement of *Sm*PncA

Data quality of *Sm*PncA was checked by the program *Xtriage* [24] of *PHENIX* software package. The crystal belongs to the space group *P*2_1_2_1_2_1_ with cell dimensions of a = 76.49 Å, b = 80.12 Å, c = 130.96 Å. *Sm*PncA contains 183 amino acid residues with a molecular weight of 20.5 kDa. There are four molecules per asymmetric unit (ASU) with a Matthews coefficient of 2.45 and solvent content of 49.8%.

**Figure 2:**
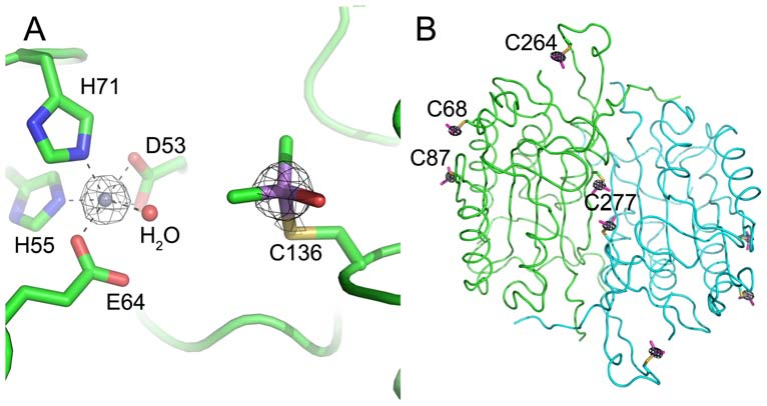
Anomalous difference Fourier map calculated with final phases at 5 σ. A. Atoms are shown as sticks model, and colored as follows: carbon, green; oxygen, red; nitrogen, blue; zinc, grey; arsenic, purple. B. The Casp6 structure is shown as a backbone representation. The cysteines modified by cacodylic acid are presented in the model.

**Figure 3:**
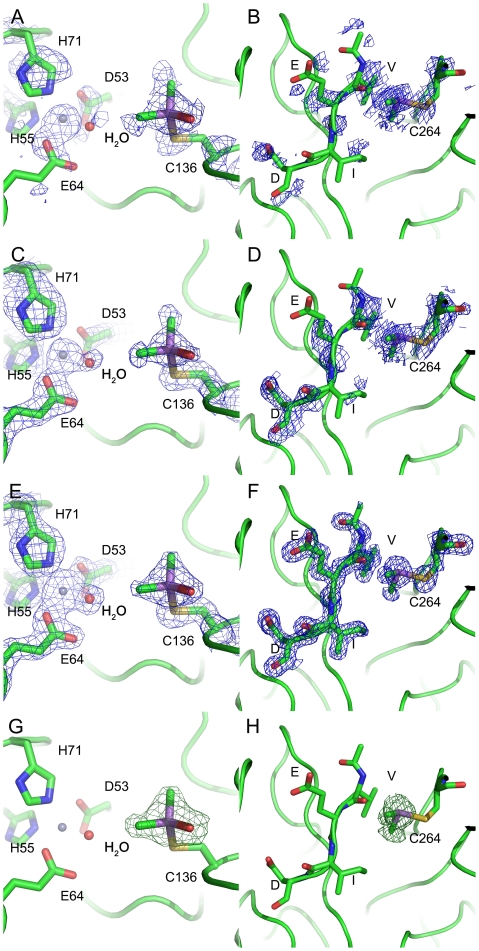
Electron density is shown around Cys136 in *sm*PncA (A,C,E,G) and Cys264 in Casp6 (B,D,F,H). A, B: SAD phases from *phaser* prior to any refinement steps. C, D: SAD phases form *resolve* after DM and NCS averaging. E, F: 2Fo-Fc map with the final phase from *phenix.refine* and G, H: Fo-Fc omit density map. This figure was generated by *PyMol*. Atoms are shown as stick and colored as follows: carbon, green; oxygen, red; nitrogen, blue; zinc, grey; arsenic, purple. The electron density is contoured at 1.5 σ, except that in the omit map (G, H), the positive density (Green) was contoured at 4 σ.

The locations of the heavy atom sites were determined by the program *SHELXD* [25]. The *phenix.autosol* in the *PHENIX* software package [26] was used to refine the locations of substructures, calculate the initial phases, and make Density Modification (DM), Non-Crystal Symmetry (NCS) improvement and model auto-building. After the initial model auto-building, 661 of total 728 residues were successfully built and 610 residues were correctly placed. The final model was manually completed by the program *Coot* [27] and further refined by *phenix.refine* in the *PHENIX* software package. The graphics program *PyMOL* [28] was used in the structural analysis and production of the figures. Anomalous difference Fourier map calculated with phases from the final model showed two obvious peaks in one monomer above 5 σ (Fig. 2A). Electron density is show around Cys136 in *sm*PncA (Fig. 3A, 3C, 3E, 3G). The coordinates and structure factors of *Sm*PncA have been deposited in the Protein Data Bank with the PDB ID code 3S2S.

### Caspase-6

#### Protein preparation, crystallization, data collection and structure information

Wild type human Casp6 were prepared as previously published [27]. Briefly, the Casp6 construct was cloned into the pET21b expression vector with a C-terminal (His)_6_-tag and expressed in *E. coli* Rosetta(DE3) strain at 18°C for 20 hours. Proteins for crystallization were purified in two steps, nickel chelating column and gel filtration chromatography, and 10mM DTT was added to the purified proteins. In order to obtain completely activated Casp6, 1 ng/µL active Casp3 was added into 5 µg/µL purified Casp6 at 4°C overnight. The Casp6 crystals were grown by the sitting drop vapor diffusion method with an inhibitor Ac-VEID-CHO added. Crystals were obtained in 20% w/v PEG 8000, 0.2 M magnesium acetate, and 0.1 M sodium cacodylate pH 6.5 at 20°C.

Diffraction data were collected on the Beamline BL6A at the Photon Factory, Tsukuba, Japan. The crystal was flash frozen in liquid nitrogen and then maintained on the goniometer at 100 K in a stream of cold nitrogen. The data were collected from one single crystal at wavelength 1.000 Å, and 180 frames were collected with 1° oscillation per image. The data were indexed, integrated by the program *Mosflm* [29] and scaled by program *Scala* [30] of the *CCP4* software package [31]. The statistics of these data are also summarized in Table 1.

#### SAD phasing for Casp6

The crystal structure of human Casp6 was first determined by Molecular Replacement method [32]. The molecular weight of Casp6 monomer is about 32 kDa and there is a homodimer in the ASU. In the structure, several cysteines on the surface were found covalently bound to cacodyl, a reductive product of cacodylate in the presence of DTT [18], [21] (Fig. 2B). We then made an attempt to make use of the As anomalous signals to determine the structure *de novo*. The data of Casp6 were re-scaled as SAD data by the Program *Scala*. The raw Bijvoet differences contributed by cacodyl were calculated to be 1.9% by the Hendrickson formulation [33]. The SAD phasing procedure of Casp6 was identical to that of *Sm*PncA. After the initial model building, about 167 residues were built into the electron density and the overall figure of merit (FOM) was 27%. Some obvious secondary structural fragments could be observed in the phase improved map. The initial model was then input into the program *phenix.autobuild* [26], another seven rounds of model auto-building were executed, after which 466 residues were auto-built. In this case, the As-SAD phasing could be used to solve the structure of Casp6 independently without any information from other models. Anomalous difference Fourier map calculated with phases from the final model showed eight obvious peaks in one ASU above 5 σ (Fig. 2B). Electron density is shown around Cys264 in casp6 (Fig. 3B, 3D, 3F, 3H). The coordinates and structure factors of casp6 have been deposited in the Protein Data Bank with the PDB ID code 3S70.

## Results and Discussion

### Arsenic/zinc SAD phasing of *Sm*PncA

*Sm*PncA consists of 183 residues with only one cysteine, Cys136, serving as the potential target for cacodylate modification. During the SAD phasing, eight heavy atom sites were found by *SHELXD* and confirmed by the program *Phaser* [34] in *Phenix.autosol*, and all of them were taken as arsenic atoms for phase calculation. However, according to the Matthews coefficient, there are only four molecules per ASU, indicating that there should be only four sites for the substructures. Since there was a classical zinc-finger fold structure found in the final model, we identified the other four anomalous scatterers as zinc atoms and further confirmed the existence of zinc atoms in the protein by Inductively Coupled Plasma Atomic Emission Spectrometry ICP-AES (data not shown). An anomalous difference Fourier map calculated with phases from the final model showed two obvious peaks in one monomer above 5 σ (Fig. 2A).

**Figure 4:**
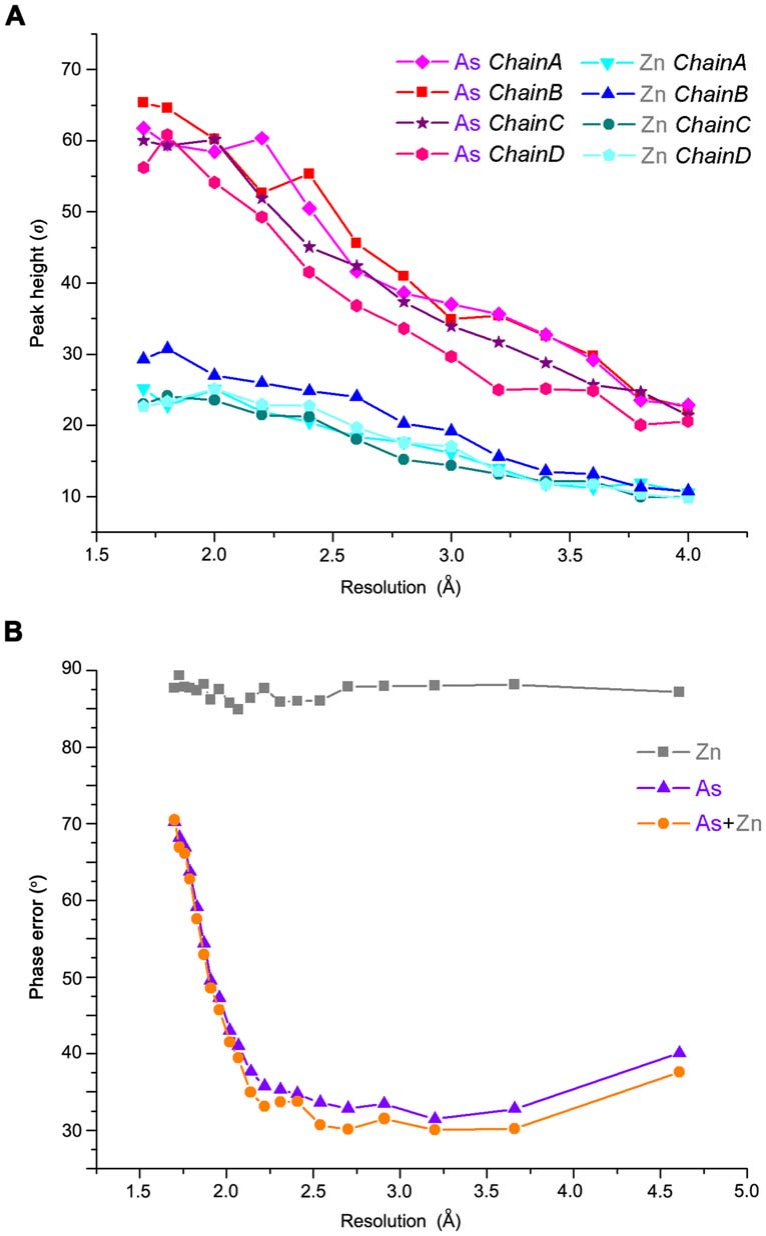
Anomalous differences between arsenic and zinc. A. The anomalous signal reduction as a function of resolution. The peak heights of the heavy atoms were derived from the anomalous Fourier map, which was calculated using final phases. B. SAD phase errors (after density modification) against the final model as a function of resolution cutoff. Different colors represent the phases calculated from different anomalous scatterers: phases calculated from zinc atoms are shown in silver, those from arsenic atoms are shown in purple and from both are shown in orange.

As the substructures solution shown in Table 2, there is a visible gap of occupancies between the two types of anomalous scatterer atoms. The occupancy of As is about 80%, whereas that of Zn is only 40%. The difference of the anomalous peak heights is consistent with the ranking in the output of program *Phaser* (Fig. 4A). All the arsenic atoms share similar anomalous peak heights, so do the zinc atoms. However, the peak heights of As are almost twice as those of Zn in the resolution range shown, indicating the phasing power of As was dominating during structure determination.

**Table 2:** Substructure solution of anomalous scatterers from *Phaser*, with each peak annotated with the corresponding atom.

Site	Occupancy from *Phaser*	Occupancy in the final model	B-factor in the final model	Atom
1	0.81	0.80	23.79	As in chain B
2	0.74	0.80	25.02	As in chain A
3	0.81	0.80	23.72	As in chain C
4	0.78	0.80	24.59	As in chain D
5	0.45	0.80	25.46	Zn in chain B
6	0.41	0.80	28.05	Zn in chain D
7	0.40	0.60	25.10	Zn in chain C
8	0.37	0.80	29.15	Zn in chain A

To test the independent phasing power of As and Zn in *Sm*PncA, the two types of heavy atoms were input into the same program pipeline separately. Program *Solve* [35] in *phenix.autosol* was used to calculate the initial phases. Further data improvement and model-building were carried out by the *phenix.autosol* pipeline. The improved phases before model-building were compared with the phases calculated from the final model, and phase errors were estimated (Fig. 4B). The model generated by As-SAD phasing consisted of 558 residues, and the remaining residues could be easily identified and placed as well. By contrast, zinc atom alone could not provide enough anomalous phasing power to generate a reasonable density map for further model building. Besides, the phase error plot clearly shows that the arsenic scatterers made the major contribution to SAD phasing in this case (Fig. 4B).

### Arsenic Modification of Cysteine in *Sm*PncA and Casp6

Most of experienced protein crystallographers often encounter unexpected anomalous scatterers during structure determinations, and these adventitious anomalous scatterers may be unverified metal ions, additive ions or molecules from the crystallization conditions. Arsenic atom sometime presents one of these kinds of anomalous scatterers. In both *Sm*PncA and Casp6, arsenic atoms were incorporated into proteins by chemical reaction between cysteines and cacodylic acid. Cacodylic acid is an As (V) compound, widely used as buffering agent in protein purification and crystallization screenings. It has a pK_a_ of 6.3, and its most effective buffering range is between 5.0 and 7.5. During crystallization, cacodylic acid can specifically modify the cysteine residues on protein surface and form a covalent bond between As and cysteine sulfur.

In the case of Casp6, proteins were prepared with DTT to avoid the formation of disulfide bond between free cysteines. When protein solution containing DTT was mixed with the crystallization reservoir, cacodylic acid was spontaneously reduced to a chemically more active As (III)-thiolate intermediate. The As (III) reagent in turn reacted with cysteines by a thiol exchange process, resulting in the formation of a cacodyl-cysteine product [18]. In this case, four out of ten cysteines in the protein covalently bound to As, while the other six cysteines were either solvent inaccessible or occupied by the inhibitor Ac-VEID-CHO in the active site.

**Figure 5:**
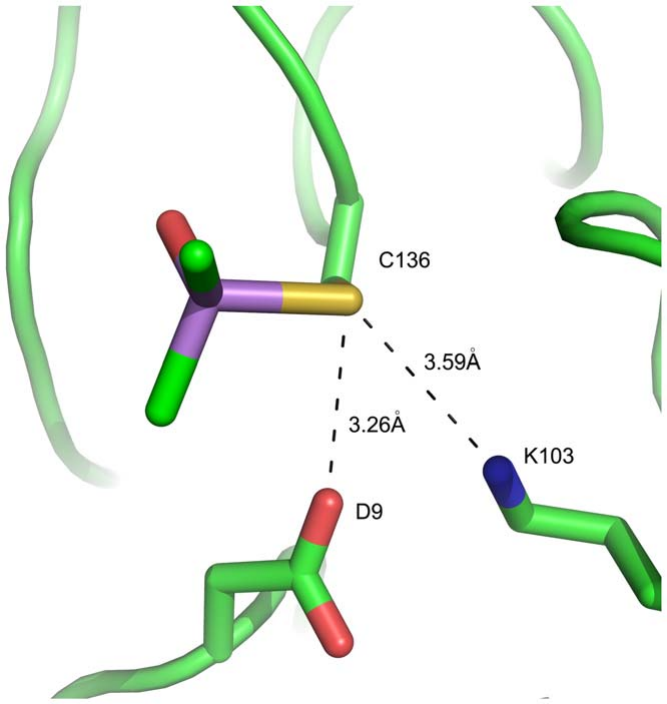
A cysteine in the *Sm*PncA is shown covalently bound to cacodylate without adding reducing agents.

**Figure 6:**
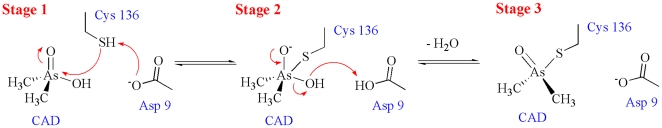
A putative three-stage cysteine modification mechanism for *Sm*PncA. In stage 1, proton abstraction by Asp9 would increase the nucleophilicities of Cys136 and facilitate nucleophilic attack at cacodylic acid (CAD); Proton donation from Asp9 to the hydroxyl group of the CAD would promote As-O bond cleavage and release of H_2_O as the intermediate in stage 2 collapses to give a dimethylarsinoyl fixed Cys136 in stage 3.

In the case of *Sm*PncA, there is no reducing agent involved in the protein preparation or crystallization. Cysteines were in contact with dimethylarsinoyl, an As (V) group. Organic As (V) reagents usually do not react effectively with organic thiols. However, surrounding residues, such as aspartate or histidine, can catalyze the thiol exchange reaction between As (V) and cysteines (Fig. 5). Similar phenomena were also found in other examples such as *Ab*PncA [36], a putative nitrilase superfamily protein [37] and a pteridine reductase [38]. Here we propose a plausible three-stage cysteine modification mechanism for *Sm*PncA without DTT (see Fig. 6), involving the Cys136 as a nucleophile to attack at arsenic in the cacodylate, and Asp9 as general acid to donate a proton.

### Comparison of arsenic with traditional phasing methods

The covalent binding of As to cysteine makes As easier to reach high occupancy in proteins compared with those non-covalent binding metals. In *Sm*PncA, the stark difference in anomalous contributions between As and Zn in phasing power is mainly due to the difference in heavy-atom incorporation manners, although the f'' of As and Zn are slightly different (at 1.000 Å wavelength, f'' (As) is 3.7e and f'' (Zn) is 2.7e). Compared with the coordination bonds between Zn and surrounding residues, the covalent bond between arsenic and cysteine is more stable and tend to generate high-occupancy site in the substructure determination. Therefore, it is more effective to use covalently bound As as anomalous scatterers than traditional soaked heavy atoms or metal scatterers in metalloproteins.

Some previous studies also showed that As modification could be an important complementary method to Se-Met substitution method in SAD phasing. In the case of SPR14 protein [16], only the combined anomalous signals from both Se-Met and As were able to produce initial phases for structural determination. In some cases, As-SAD phasing is more advantageous than Se-SAD phasing, such as the structure determinations of HIV-1 integrase [17], [21]. Se-Met substitution method is quite expensive and time-consuming, usually gives lower protein expression level, and not always works well for recombinant proteins in eukaryotic expression systems. By contrast, the reaction between cacodylate and free cysteines on protein surface can be easily handled without extra steps during protein expression and purification.

In the case of *Sm*PncA, one As anomalous scatterer could phase up to 200 amino acid residues. According to statistical analyses, the occurrence of cysteines in proteins is about 2% [39], [40], [41], therefore, if one out of four cysteines could be solvent-accessible and modified by As, many proteins would have fairly good chances to be successfully phased by As-SAD. In addition to the potential phasing capacity, surface-cysteine modification by arsenic compound could be beneficial to protein crystallization as well [42], [43]. Modified surface cysteines are not able to form unfavorable disulfide bonds, and the small arsenic compound will not bring in significant disturbance to protein structures.

The reaction of cacodylic acid with cysteine has been observed many times before. According to the statistical data, more than eighty protein structures in the PDB database have As-modified cysteines. However, this reaction has not been notified to serve as an effective and general method to introduce As into proteins. Based on our studies, we propose that the cacodylate buffer can be used to crystallize proteins that have free surface cysteines.

In conclusion, we have solved the crystal structures of two proteins by As-SAD phasing method and further studied the details of arsenic incorporation by the reaction between cysteine and cacodylate, the buffering agent in the crystallization conditions. We would like to suggest to the protein crystallography community that cacodylate buffer can be used to introduce arsenic simply into proteins for SAD phasing in general.
